# Case Report: Complete hydatidiform mole mimicking spondyloarthritis: a probable paraneoplastic rheumatologic syndrome

**DOI:** 10.3389/fimmu.2026.1866294

**Published:** 2026-05-29

**Authors:** Zhaomeng Gao, Qin Zhang, Zhenzhen Ma

**Affiliations:** 1 Department of Rheumatology and Immunology, Shengli Oilfield Central Hospital, Dongying, Shandong, China; 2 School of Biomedicine and Nursing, Shandong Institute of Petroleum and Chemical Technology, Dongying, Shandong, China

**Keywords:** complete hydatidiform mole, gestational trophoblastic disease, paraneoplastic syndrome, rheumatologic manifestation, spondyloarthritis, β-human chorionic gonadotropin

## Abstract

**Background:**

Complete hydatidiform mole (CHM) is a gestational trophoblastic disease characterized by abnormal proliferation of trophoblastic tissue and markedly elevated β-human chorionic gonadotropin (β-hCG) levels. Although CHM typically presents with vaginal bleeding and uterine enlargement, it may occasionally manifest with paraneoplastic systemic symptoms that mimic autoimmune rheumatic diseases.

**Case presentation:**

A 33-year-old woman presented with a 2-month history of polyarthritis, inflammatory low back pain, and morning stiffness, initially suspected as spondyloarthritis (SpA). She had a history of missed miscarriage at 7 weeks of gestation with medical abortion two months prior. Physical examination revealed dactylitis of the left third finger and right fifth toe, enthesitis at bilateral heels, and tenderness over the lumbar spine. Laboratory tests showed elevated erythrocyte sedimentation rate (50 mm/h) and C-reactive protein (42.8 mg/L). HLA-B27 was positive, while rheumatoid factor, anti-cyclic citrullinated peptide antibodies, and antinuclear antibodies were negative. Pelvic ultrasonography revealed a heterogeneous echogenic intrauterine mass (4.8 × 4.3 × 3.6 cm) with a “honeycomb” or “snowstorm” appearance, suggestive of gestational trophoblastic disease. Serum β-hCG was markedly elevated at 31,870 IU/L. Bone scintigraphy demonstrated increased uptake in the left wrist, metacarpophalangeal joints, interphalangeal joints, left ankle, and L1 spinous process. Uterine curettage was performed, and histopathology confirmed complete hydatidiform mole. Following the procedure, arthralgia and back pain resolved dramatically without disease-modifying antirheumatic drugs or biologics. Serum β-hCG declined progressively to normal levels, and inflammatory markers normalized. At 2-year follow-up, the patient remained asymptomatic, and repeat bone scintigraphy showed no abnormal uptake.

**Conclusion:**

This case highlights that CHM can present with rheumatic manifestations mimicking SpA, including inflammatory back pain, peripheral arthritis, dactylitis, enthesitis, and even HLA-B27 positivity. The parallel course of rheumatic symptoms with β-hCG levels, rapid resolution after uterine evacuation, and sustained remission during follow-up are consistent with a probable paraneoplastic rheumatologic syndrome rather than primary SpA. Clinicians should maintain a high index of suspicion for gestational trophoblastic disease in women of childbearing age presenting with new-onset inflammatory arthritis and elevated β-hCG.

## 1 Introduction

Spondyloarthritis (SpA) encompasses a group of chronic inflammatory rheumatic diseases characterized by axial skeleton involvement, sacroiliitis, peripheral arthritis, enthesitis, and dactylitis (1). The diagnosis relies on a combination of clinical features, imaging findings, and HLA-B27 positivity. However, several paraneoplastic syndromes, particularly those associated with lymphoma and multiple myeloma, can mimic SpA and pose significant diagnostic challenges (2, 3).

Complete hydatidiform mole (CHM) is the most common form of gestational trophoblastic disease, characterized by hydropic villi and diffuse trophoblastic hyperplasia. It typically presents with vaginal bleeding, uterine enlargement, and excessively elevated β-human chorionic gonadotropin (β-hCG) levels (4). Rarely, gestational trophoblastic diseases, including CHM, may induce paraneoplastic systemic manifestations, including hyperthyroidism, ovarian theca lutein cysts, and, exceptionally, lupus-like autoimmune syndromes (5–8). The pathophysiology likely involves the pro-inflammatory cytokine cascade and potential molecular mimicry driven by elevated β-hCG or associated trophoblastic factors (9, 10).

Here, we report a challenging case of CHM that presented with inflammatory back pain, peripheral arthritis, dactylitis, and enthesitis, initially misdiagnosed as SpA. The rheumatic manifestations resolved completely following uterine curettage, underscoring the importance of considering gestational trophoblastic disease in the differential diagnosis of new-onset inflammatory arthritis in women of reproductive age.

## 2 Case presentation

A 33-year-old woman was admitted to our department on November 23, 2023, complaining of progressive polyarticular swelling and pain accompanied by lower back pain for two months, with exacerbation over the preceding week.

### 2.1 History of present illness

Two months prior to admission (September 2023, at 7 weeks of gestation), the patient developed swelling and pain in the left third proximal interphalangeal joint and bilateral ankles, with sausage-like swelling (dactylitis) of the left third finger. She also experienced persistent lower back pain, significant morning stiffness, and nocturnal pain that interfered with sleep and notably, the pain did not improve with activity, which is atypical for classic inflammatory back pain and contributed to the initial diagnostic confusion. No special treatment was sought initially. At approximately 50 days of gestation, vaginal bleeding occurred; ultrasonography revealed abnormal embryonic morphology, and a medical abortion was performed. However, mild vaginal bleeding persisted post-abortion.

**Figure 1:**
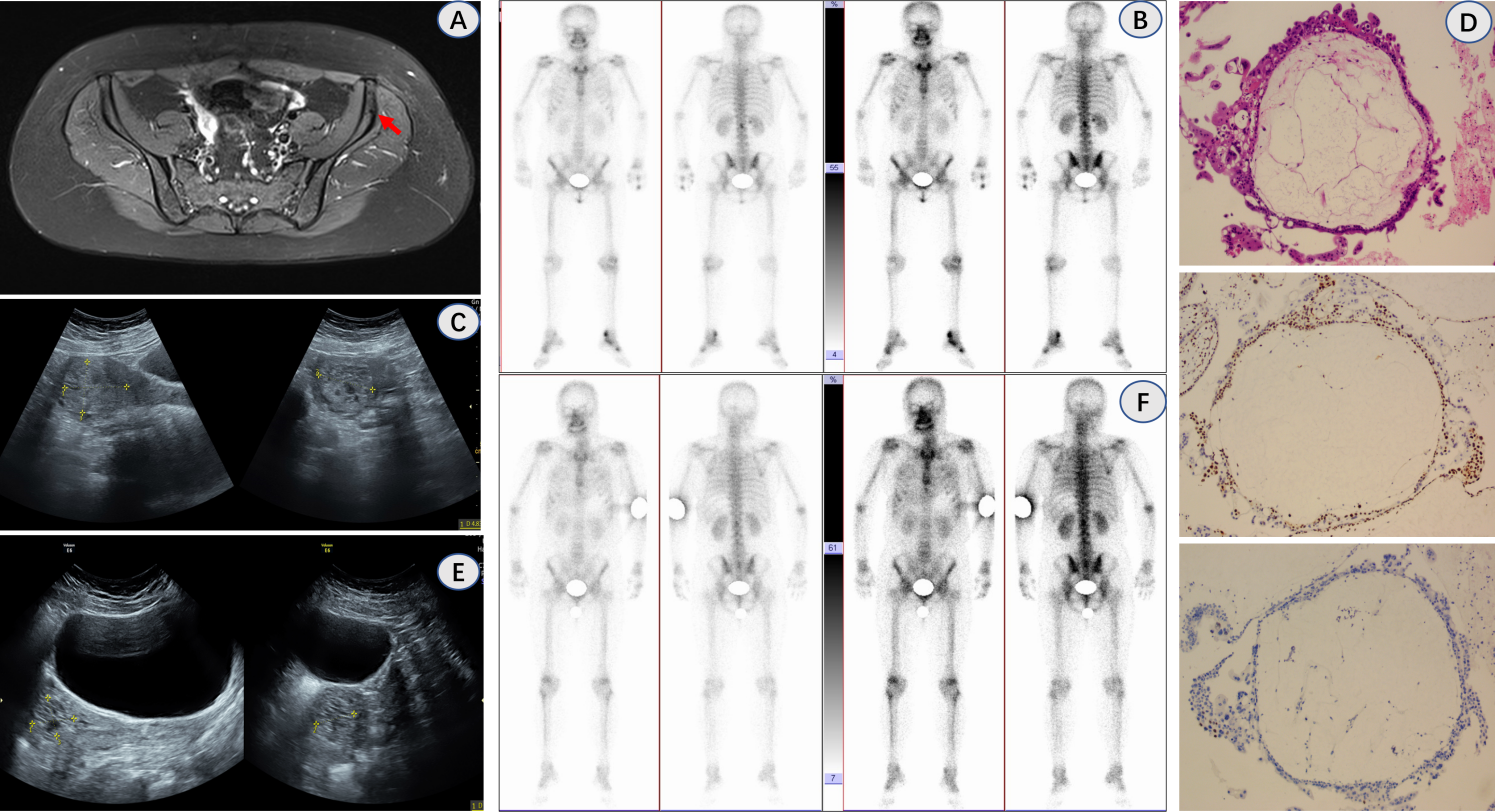
Imaging findings during the disease course. **(A)** Sacroiliac joint MRI (November 2023): Small cystic lesion in the left ilium (red arrow) with preserved joint space and no sacroiliitis. **(B)** Bone scintigraphy (November 2023): Increased uptake in left wrist, metacarpophalangeal joints, interphalangeal joints, left ankle, and L1 spinous process. **(C)** Pelvic ultrasonography (November 2023): Intrauterine heterogeneous honeycomb mass (4.8 × 4.3 × 3.6 cm) suggestive of gestational trophoblastic disease. **(D)** Histopathology of first curettage (December 2, 2023): Curettage specimen showing hydropic villi with cistern formation and circumferential trophoblastic hyperplasia (H&E, ×100), accompanied by high Ki−67 expression and loss of nuclear p57 in cytotrophoblasts and villous stroma, confirming complete hydatidiform mole. **(E)** Pelvic ultrasonography (December 5, 2023): Residual honeycomb echogenic lesion in uterine cavity after first curettage. **(F)** Follow-up bone scintigraphy (December 2025): Complete resolution of previously noted abnormal uptake.

On October 8, 2023, laboratory testing revealed a CRP of 61.3 mg/L, and ESR 64 mm/h. Ultrasonography indicated tenosynovitis of the left third finger. After treatment with loxoprofen sodium, joint pain partially improved. Nevertheless, the disease progressed to involve the bilateral ankles, left knee, bilateral shoulders, left first metacarpophalangeal joint, left thumb interphalangeal joint, right fifth metatarsophalangeal joint and interphalangeal joint, and bilateral heels, with severe pain and sausage-like swelling of the right fifth toe. Outpatient workup showed positive HLA-B27, while antinuclear antibodies (ANA), anti-cyclic citrullinated peptide (CCP) antibodies, anti-keratin antibodies (AKA), anti-perinuclear factor (APF), and rheumatoid factor (RF) were all negative. Magnetic resonance imaging (MRI) of the sacroiliac joints demonstrated a small cystic lesion in the left ilium (well-defined, low T1 and high T2 signal) with preserved joint spaces and no abnormal soft tissue signals (**Figure 1A**). Treatment was initiated with intermittent diclofenac sodium (taken only during periods of significant pain), sulfasalazine 1 g twice daily (continued for approximately 1 month), methotrexate 15 mg once weekly (continued for approximately 1 month), and methylprednisolone 12 mg once daily (used for 1 week only). Symptomatic relief was partial and fluctuating; notably, joint pain and swelling worsened 1 week before admission despite this regimen, accompanied by neck pain and new involvement of the left third metacarpophalangeal joint and left wrist.

### 2.2 Past medical and obstetric history

She denied tobacco smoking and alcohol consumption. The patient had a gravidity of 5 and parity of 1. Her obstetric history included one missed miscarriage at 2 months of gestation, two induced abortions for unplanned pregnancies, and one medical abortion at 50 days of gestation due to abnormal embryonic morphology with vaginal bleeding (the index pregnancy). She denied any history of psoriasis, inflammatory bowel disease, uveitis, or family history of SpA.

### 2.3 Physical examination on admission

The patient appeared distressed. Tenderness was elicited over the L1–L2 vertebral bodies. Swelling and tenderness were noted at the left third proximal interphalangeal joint, left first and third metacarpophalangeal joints, left thumb interphalangeal joint, left ankle, right fifth metatarsophalangeal and interphalangeal joints, and bilateral heels. Tenderness without overt swelling was present at the left knee and left shoulder.

### 2.4 Laboratory and imaging investigations

On November 24, 2023, laboratory tests revealed an erythrocyte sedimentation rate (ESR) of 50 mm/h (reference range: 0–20 mm/h) and CRP of 42.8 mg/L (reference range: 0–6 mg/L). Complete blood count, liver and renal function, antinuclear antibody profile, antiphospholipid antibodies, immunoglobulins, and complement levels were all within normal limits. Notably, serum β-hCG on November 27, 2023, was markedly elevated at 31,870 IU/L (non-pregnant reference: 0–5 IU/L). Bone scintigraphy (technetium-99m methylene diphosphonate, 99mTc-MDP) performed on November 25, 2023, showed increased radiotracer uptake in the left wrist, left metacarpophalangeal joints, left interphalangeal joints, left ankle, and L1 spinous process, suggestive of active arthritis and enthesitis, although bone scintigraphy is nonspecific for definitive diagnosis. No other abnormal uptake or photopenic lesions were identified (**Figure 1B**). Transvaginal ultrasonography on November 26, 2023, revealed an intrauterine heterogeneous echogenic mass measuring 4.8 × 4.3 × 3.6 cm with a honeycomb appearance, poorly defined margins relative to the myometrium, and peripheral dot-like blood flow signals on color Doppler flow imaging (CDFI). The report suggested gestational trophoblastic disease (**Figure 1C**).

### 2.5 Clinical course and treatment

The preliminary diagnoses were: (1) possible spondyloarthritis; and (2) gestational trophoblastic disease. The patient was treated symptomatically with diclofenac sodium sustained-release tablets and technetium [99Tc] methylenediphosphonate injection (a therapeutic radiopharmaceutical used for inflammatory arthritis in China). A gynecology consultation was obtained, and repeat β-hCG testing together with uterine curettage was recommended.

On December 1, 2023, β-hCG was 32,650 IU/L. The first uterine curettage was performed on December 2, 2023. Histopathological examination confirmed complete hydatidiform mole (**Figure 1D**). p57 immunohistochemistry showed complete loss of nuclear staining in villous cytotrophoblasts and intermediate trophoblastic cells, with preserved nuclear staining in maternal decidual cells serving as internal positive controls. Ki-67 demonstrated diffuse strong positivity (>90%). Molecular genotyping or ploidy analysis was not performed at our institution, and the diagnosis relied on morphological and immunohistochemical criteria. Postoperatively, the patient reported significant relief of joint swelling, pain, and lower back pain. Diclofenac was gradually tapered.

Follow-up β-hCG on December 5, 2023, was 23,389 IU/L, and pelvic ultrasonography still showed a honeycomb echogenic lesion within the uterine cavity (**Figure 1E**). On December 10, 2023, β-hCG was 23,713 IU/L. A second curettage was performed on December 20, 2023, with histopathology again confirming complete hydatidiform mole. After the second procedure, joint symptoms further resolved, and back pain disappeared.

By December 23, 2023, pelvic ultrasonography showed no obvious intrauterine abnormality. On December 24, 2023, β-hCG had declined to 929.93 IU/L. Thyroid function tests were performed: TSH, free T3, and free T4 were all within normal limits, with no evidence of hyperthyroidism or hypothyroidism. The patient was discharged. During outpatient follow-up, β-hCG progressively normalized, and joint symptoms and back pain completely resolved. ESR and CRP returned to normal ranges. All medications, including non-steroidal anti-inflammatory drugs, were discontinued.

**Figure 2:**
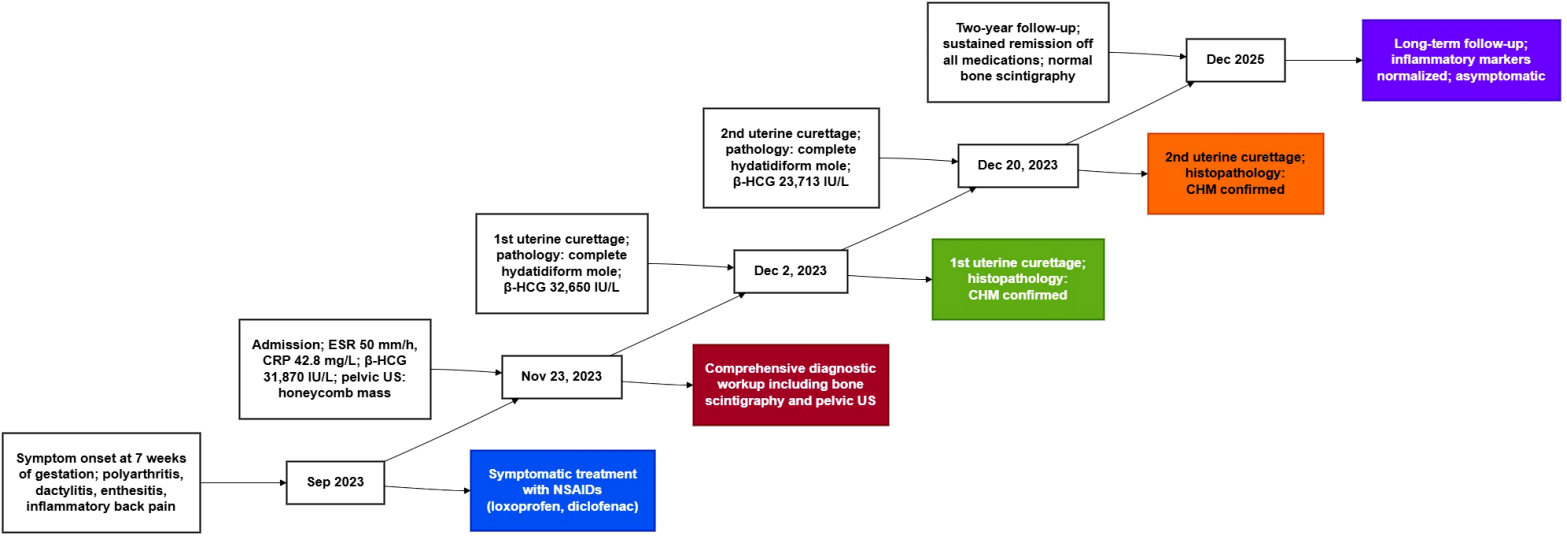
Clinical timeline from symptom onset to 2-year follow-up. Upper panel: Key clinical milestones. Lower panel: Management phases. The patient presented with inflammatory arthritis at 7 weeks of gestation (September 2023), was diagnosed with complete hydatidiform mole following two uterine curettages (December 2 and 20, 2023), and achieved sustained remission off all medications at 2-year follow-up (December 2025). CHM, complete hydatidiform mole; ESR, erythrocyte sedimentation rate; CRP, C-reactive protein; β-hCG, β-human chorionic gonadotropin; US, ultrasonography; NSAIDs, non-steroidal anti-inflammatory drugs.

At 2-year follow-up (December 2025), the patient remained free of arthralgia, back pain, or morning stiffness. Repeat bone scintigraphy demonstrated no abnormal radiotracer uptake (**Figure 1F**). The clinical timeline from symptom onset to 2-year follow-up is summarized in **Figure 2**, and detailed chronological data are provided in **Supplementary Table 1**.

### 2.6 Gynecologic follow-up and surveillance

Following the second uterine curettage on December 20, 2023, the patient was enrolled in a structured post-molar surveillance program according to institutional protocols aligned with FIGO/WHO guidelines. Serum β-hCG was monitored weekly until three consecutive negative results were obtained, and then monthly for 6 months. The β-hCG trend was as follows: 23,713 IU/L (December 10, pre-second curettage) → 929.93 IU/L (December 24, discharge) → 47.15 IU/L (January 17, 2024) → <5 IU/L (normalized by February 22, 2024).

Post-molar gestational trophoblastic neoplasia (GTN) was rigorously excluded based on the following criteria: (1) β-hCG demonstrated a consistent exponential decline without plateau or rebound; (2) chest computed tomography performed in January 17, 2024 revealed no pulmonary metastases; (3) histopathology of the second curettage specimen showed residual hydropic villi with trophoblastic hyperplasia but no choriocarcinoma or invasive mole; and (4) no new metastatic lesions or abnormal vaginal bleeding occurred during follow-up. The patient received comprehensive contraception counseling and was advised to use reliable contraception and avoid pregnancy for 12 months. Compliance was confirmed during outpatient visits.

## 3 Discussion

This case illustrates a rare but clinically important presentation of complete hydatidiform mole masquerading as spondyloarthritis. Several key features support the diagnosis of a paraneoplastic rheumatologic syndrome rather than primary SpA.

The temporal relationship between the onset of rheumatic manifestations and gestational trophoblastic disease is consistent with a paraneoplastic process. The patient developed inflammatory arthritis and back pain at 7 weeks of gestation, and symptoms persisted following the initial incomplete medical abortion. The markedly elevated β-hCG (31,870 IU/L) and the characteristic ultrasonographic and histopathological findings established the diagnosis of CHM.

Moreover, the parallel course of rheumatic symptoms and β-hCG levels is highly suggestive of a paraneoplastic etiology. Crucially, the dramatic resolution of arthritis, enthesitis, and inflammatory back pain following uterine curettage--without the need for sustained immunosuppressive or biologic therapy--strongly indicates that the rheumatic manifestations were driven by the underlying trophoblastic pathology. The normalization of inflammatory markers and the sustained 2-year remission off all medications further argue against a chronic primary rheumatic disease.

### 3.1 Differential diagnosis

A comprehensive differential diagnosis was considered. Primary SpA was deemed unlikely because the back pain was atypical (no improvement with activity), MRI lacked definite sacroiliitis, and the disease course tracked with β-hCG levels rather than following a chronic relapsing pattern. Reactive arthritis was excluded by the absence of preceding genitourinary or gastrointestinal infection and negative bacterial cultures. Viral arthritis (parvovirus B19, HBV, HCV, HIV) was excluded by negative serology. Rheumatoid arthritis was ruled out by negative RF and anti-CCP antibodies and the absence of characteristic small-joint synovitis. Psoriatic arthritis was excluded by the lack of psoriasis or nail changes. IBD-associated arthritis was unlikely given the absence of gastrointestinal symptoms. Connective tissue disease was excluded by negative ANA/ENA and normal complement levels.

### 3.2 Comparison with previously reported cases

To our knowledge, only one prior English-language case report has described CHM mimicking SpA. Arezzo et al. (11) reported a 36-year-old woman who presented with inflammatory low back pain and MRI-confirmed bilateral sacroiliitis in the setting of a complete hydatidiform mole. In that case, the rheumatic manifestations were predominantly axial, fulfilling the imaging criteria for axial SpA, and symptoms resolved following uterine evacuation.

By contrast, our patient presented with a predominantly peripheral phenotype, characterized by asymmetric oligoarthritis, dactylitis (sausage-like swelling of the left third finger and right fifth toe), enthesitis at bilateral heels, and tenosynovitis. Although she also reported inflammatory back pain, the pain did not improve with activity, which is atypical for classic inflammatory back pain and initially contributed to diagnostic confusion. MRI of the sacroiliac joints did not demonstrate definite sacroiliitis; instead, only a small iliac cystic lesion was observed, and bone scintigraphy revealed polyarticular uptake rather than isolated sacroiliac involvement. In retrospect, this atypical feature likely reflected multifocal enthesitis—notably L1 spinous process uptake on scintigraphy—rather than primary axial inflammation, further arguing against a diagnosis of true axial SpA. Furthermore, our patient was HLA-B27 positive, which has not been reported in the previous case and adds complexity to the differential diagnosis.

The comparison between these two cases highlights an important clinical spectrum: gestational trophoblastic disease may induce paraneoplastic rheumatic syndromes that phenocopy either axial or peripheral SpA, or both. The pathophysiology likely involves a systemic inflammatory cytokine cascade driven by trophoblastic factors, with individual phenotypic expression influenced by genetic background (e.g., HLA-B27) and possibly the site of immune complex deposition or molecular mimicry.

### 3.3 Pathophysiological considerations

The pathophysiology of CHM-associated paraneoplastic rheumatic syndrome remains incompletely understood. Proposed mechanisms include: (1) the pro-inflammatory effects of excessively elevated β-hCG, which may stimulate cytokine production (e.g., tumor necrosis factor-α, interleukin-6) and activate immune cells (12, 13); (2) the trophoblastic tissue itself secretes pro-inflammatory mediators, either directly or via trophoblast-derived exosomes (12, 14, 15); and (3) the potential contribution of epitope spreading or molecular mimicry triggered by trophoblastic antigens is hypothetical and awaits experimental validation. The HLA-B27 positivity in this patient raises an important diagnostic and pathophysiological question: did the trophoblastic disease merely phenocopy SpA, or did it trigger SpA-like autoinflammation in a genetically predisposed individual? HLA-B27 is strongly associated with primary SpA, yet population studies have not reported an increased prevalence of HLA-B27 in gestational trophoblastic disease–associated rheumatic syndromes. Therefore, the HLA-B27 positivity in this case may represent either (a) an incidental genetic background with phenocopy by the trophoblastic disease, or (b) a predisposing factor that facilitated phenotypic expression of SpA-like features under the inflammatory stimulus of β-hCG and trophoblastic mediators. This distinction cannot be resolved by a single case report and warrants future registry-based or genetic association studies.

### 3.4 Limitations

Despite the compelling temporal association, several limitations should be acknowledged. First, the precise molecular mechanism linking β-hCG elevation to SpA-like synovitis remains hypothetical; existing evidence derives primarily from *in vitro* cytokine studies and lacks direct demonstration of β-hCG–mediated enthesitis in animal models. Second, the HLA-B27 positivity may represent an incidental genetic background rather than a true pathogenetic factor, as population studies have not established an increased prevalence of HLA-B27 in CHM-associated rheumatic syndromes. Third, the absence of synovial biopsy or advanced imaging (PET-CT, contrast-enhanced MRI) precludes definitive characterization of the inflammatory pattern. Future multicenter case series or registry-based studies are needed to validate this paraneoplastic association and identify predictive biomarkers.

### 3.5 Clinical implications

This case carries important clinical implications. Rheumatologists should include gestational trophoblastic disease in the differential diagnosis of new-onset inflammatory arthritis or back pain in women of reproductive age, particularly when associated with recent pregnancy loss, abnormal vaginal bleeding, or disproportionately elevated inflammatory markers. Measurement of serum β-hCG is essential in such clinical scenarios and can prevent misdiagnosis and unnecessary long-term immunosuppression.

The distinction between primary SpA and a paraneoplastic mimic is critical. In our case, several “red flags” should have raised suspicion for an alternative diagnosis: (1) the onset coincided with early pregnancy and followed an abnormal gestational event; (2) the peripheral arthritis was unusually severe and extensive despite conventional SpA therapy (sulfasalazine, methotrexate, methylprednisolone); (3) the MRI lacked definitive sacroiliitis despite prominent axial symptoms; and (4) most importantly, the disease course tracked with β-hCG levels rather than following the expected chronic relapsing pattern of SpA.

## Data Availability

The raw data supporting the conclusions of this article will be made available by the authors, without undue reservation.
