# Supplementary material for: Case Report: Complete hydatidiform mole mimicking spondyloarthritis: a probable paraneoplastic rheumatologic syndrome
Source: Front Immunol. 2026 May 29;17:1866294. doi: 10.3389/fimmu.2026.1866294 (PMC13260584; doi:10.3389/fimmu.2026.1866294)
Supplement: Supplementary file 1 [file Table1.docx]

**Supplementary Table 1. Timeline of clinical, laboratory, imaging, and therapeutic events.**

| **Date** | **β-hCG (IU/L)** | **ESR (mm/h) / CRP (mg/L)** | **Symptoms** | **Imaging** | **Treatment** | **Clinical Response** |
| --- | --- | --- | --- | --- | --- | --- |
| Sep 2023 | — | — | Polyarthritis,  back pain,  morning stiffness | — | — | — |
| Oct 8, 2023 | — | ESR 64;  CRP 61.3 | Progressive polyarthritis | US: tenosynovitis  (L 3rd finger) | Loxoprofen sodium | Partial relief |
| Nov23–27, 2023 | 31,870 | ESR 50;  CRP 42.8 | Worsening;  neck pain;  new wrist involvement | MRI SI joints: cystic lesion (L ilium);  Bone scintigraphy: uptake (L wrist, MCP, IP, ankle, L1);  TVUS: honeycomb mass (4.8×4.3×3.6 cm) | Intermittent diclofenac,  SSZ 1 g bid,  MTX 15 mg qw, methylprednisolone 12 mg qd | Partial, fluctuating |
| Dec 1, 2023 | 32,650 | — | — | — | — | — |
| Dec 2, 2023 | — | — | — | — | First uterine curettage | Significant relief |
| Dec 5, 2023 | 23,389 | ESR 46;  CRP 32.5 | Improved | TVUS: residual intrauterine lesion | - | Improved |
| Dec 10, 2023 | 23,713 | — | — | — | — | — |
| Dec 20, 2023 | — | — | — | — | Second uterine curettage | Back pain resolved |
| Dec 24, 2023 | 929.93 | ESR 27;  CRP 16.9 | Asymptomatic | TVUS: no abnormality; TFT: normal | Discharged; all medications discontinued | Dramatic resolution |
| Jan 17, 2024 | 47.15 | Normalizing | Asymptomatic | Chest CT: no metastasis | — | GTN excluded |
| Feb, 22，2024 | <5 | Normal | Asymptomatic | — | — | β-hCG normalized |
| Dec，2025 | <5 | Normal | Asymptomatic | Bone scintigraphy: no abnormal uptake | None | 2-year sustained remission |

Abbreviations: β-hCG, β-human chorionic gonadotropin; ESR, erythrocyte sedimentation rate; CRP, C-reactive protein; US, ultrasonography; MRI, magnetic resonance imaging; SI, sacroiliac; L, left; MCP, metacarpophalangeal; IP, interphalangeal; TVUS, transvaginal ultrasonography; TFT, thyroid function test; CT, computed tomography; GTN, gestational trophoblastic neoplasia; SSZ, sulfasalazine; MTX, methotrexate; qd, once daily; qw, once weekly; bid, twice daily; PRN, as needed.
